# How can we attain enhanced quality assurance of the mode of birth?

**DOI:** 10.18332/ejm/152157

**Published:** 2022-08-04

**Authors:** Paraskevi Giaxi, Aikaterini Lykeridoy, Victoria G. Vivilaki

**Affiliations:** 1 Department of Midwifery, School of Health and Care Sciences, University of West Attica, Athens, Greece

**Keywords:** quality, Robson classification, childbirth, cesarean section, indicators

The concept of quality in healthcare is a core issue that gets ever more interesting and important. According to WHO, the definition of quality in care is ‘the extent to which healthcare services provided to individuals and patient populations improve desired health outcomes. In order to achieve this, healthcare must be safe, effective, timely, efficient, equitable and people-centred’^1^. Thus, as quality of healthcare for women and newborns, we define the extent to which the provided services, whether individual or not, meet the requirements to achieve the desired outcomes, that are, at the same time, in line with contemporary practices and accommodate the patients’ needs and desires^2^.

The emphasis on securing quality in care led to the measurement of indicators^2-4^. Quality indicators serve as ‘measurement tools that can be used to monitor, evaluate and improve the quality of patient care, organization and support services that affect patient outcomes’^5^. The results of these indicators are the starting point for achieving accountability and quality improvement in healthcare by involving all sectors of the health system in this endeavour^6^. Indicators such as cesarean section rate, cesarian section rate before labor, cesarian section rate during labor, cesarean section rate before labor in low-risk women, cesarean section rate during labor in low-risk women, proportion of women with induced labor with an indication of post-dates who are less than 41 weeks of gestation at delivery, the ratio of women with labor induction who give birth after 41 weeks of pregnancy, the proportion of pregnant women having a planned cesarean section who have the procedure carried out at or after 39 weeks 0 days, rate of recurring cesarean section in low-risk women before 39 weeks gestation, instrumental vaginal delivery rate, have been recommended and adopted by researchers^6,7^. As the World Health Organization acknowledges the significantly increased rates of cesarian sections as a worldwide phenomenon, as well as the difficulties that arise worldwide in interpreting these rates, they recommend the use of the Robson classification as a ‘global standard for assessing, monitoring and comparing cesarean section rates within healthcare facilities over time, and between facilities’^8^. The Robson classification incorporates epidemiological variables and is a standardized tool for monitoring and comparing cesarian section rates. It can be used worldwide and is helpful to clinicians, facility administrators, public health authorities and women themselves and is characterized as simple, accountable, replicable, clinically relevant, retrospective, and verifiable.

Studying birth allocation by Robson classification has been a scope of application and study in many countries^9^. In February 2021, acknowledging the importance of adopting the Robson scale, Euro-Peristat published complementary data from the published report on perinatal health in 2015 that included only indicators. Data on the use of the Robson classification were provided by 55% of the countries, with 18 out of 31 countries providing comprehensive recording data^10^. Rising cesarean section rates require countries to implement evidence-based strategies to reduce those that are unnecessary. Ministries of Health, health authorities and hospital managers should recommend the implementation of the Robson classification in clinical audits to improve the quality of health services provided.

## Data Availability

Data sharing is not applicable to this article as no new data were created.
